# A Successful Gap-Year Clinical Research Technician (CRT) Program at an Academic Anesthesiology Department

**DOI:** 10.7759/cureus.39000

**Published:** 2023-05-14

**Authors:** Anusha N Samant, Jessica E Fanelli, Ashish K Khanna, Scott Segal

**Affiliations:** 1 Anesthesiology, Wake Forest University School of Medicine, Winston-Salem, USA; 2 Anesthesiology, Tulane University School of Medicine, New Orleans, USA; 3 Anesthesiology, Wake Forest School of Medicine, Winston-Salem, USA

**Keywords:** research resources, medical school, gap year, graduate health school, educational tool, clinical research technician

## Abstract

Background: Many students elect to take gap years in between graduating college and matriculating into medical school. At an academic institution, investigators can be limited in conducting research due to clinical endeavors. Utilizing a structured, clinical research, gap-year program with students called clinical research technicians (CRTs) can aid both investigators conducting research and students applying to graduate health programs. In this original article, we sought to understand CRT and investigator perceptions of and experiences in the program.

Methods: We distributed a survey to past and present CRTs and the investigators with whom they worked at Atrium Health Wake Forest Baptist Medical Center. We conducted thematic and sentiment analyses of the survey results. We also collected data on grant approvals, research funding awards, and CRT, clinical research nurse, and clinical research coordinator salaries.

Results: We received responses from 20/29 investigators and 21/22 CRTs. We identified five themes for the investigator survey, including research accuracy and precision; research output; alleviating responsibilities; cost; and likelihood of referral. We identified five themes for the CRT survey, including future career assistance; physician career insights; mentorship; likelihood of referral; and other. The majority of respondents strongly agreed or agreed with the survey statements. The majority of comments were coded as positive. All of CRTs were accepted into a graduate health profession program.

Conclusions: Our program’s success demonstrates how a structured, clinical research, gap-year program for premedical students can serve as a new educational tool and important research infrastructure resource for hospitals.

## Introduction

Many pre-medical students are electing to take gap year(s) in between graduating college and matriculating into medical school. In the past five years, the number of pre-medical students reporting that one year or more had passed since graduating college before starting medical school increased from 63.4% to 71.1% [1]. There are many reasons why pre-medical students elect to take a gap-year such as wanting to work/volunteer to gain more experience to strengthen their application, taking courses to meet premedical requirements, or being waitlisted or denied admission, requiring reapplication in the next academic cycle. However, some of the most popular uses of time during a gap-year include working at another career or working/volunteering in a healthcare setting or research [1-2]. These experiences can provide insight into the intricacies of a career in healthcare without the added stressors associated with a formal medical education.

One such opportunity is the clinical research technician (CRT) program, which employs gap-year students to assist investigators conducting research projects in the Anesthesiology Department at the Atrium Health Wake Forest Baptist Medical Center. The program started in 2016 with a single CRT and has since grown to 13 CRTs in the latest cohort. Investigators often face limitations in their research output due to time-consuming clinical responsibilities [3]. Utilizing other research personnel, such as research nurses, research fellows, and clinical research coordinators, can help lessen the burden of conducting research and increase academic output. However, this can be costly to the department. The CRT program aims to alleviate this burden while also providing gap-year students with insights into the healthcare field and improving their chances of successful matriculation into graduate health programs.

In this study, we investigate the effectiveness and impact of the CRT program. Specifically, we aim to: 1. Understand CRT and investigator perceptions of and experiences in the program. 2. Assess the program's success in aiding gap-year students in matriculating to medical school and being prepared for clinical interactions. 3. Compare the cost of using CRTs versus clinical research nurses or clinical research coordinators to conduct research. 4. Evaluate the program's impact on research grant approval and funding since its inception. To achieve these objectives, we distributed a survey to past and present CRTs and the investigators who utilized the help of CRTs. We also collected data on grant approvals, research funding awards, and CRT, clinical research nurse, and clinical research coordinator salaries. Through our analysis, we aim to demonstrate the value of a structured, clinical research, gap-year program for premedical students as a new educational tool and important research infrastructure resource for hospitals.

## Materials and methods

This study was an opt-in survey of past and present CRTs and investigators with whom they worked. Investigators refers to any physician or faculty member within the Department of Anesthesiology who has or is currently conducting research. The protocol was approved by the Wake Forest University Health Sciences’ Institutional Review Board (Winston-Salem, North Carolina) and the survey was deemed exempt, so no informed consent was obtained. The research team included the Head CRTs for the 2021-2022 and 2022-2023 cohorts, the Department Vice Chair of Research, and Chair of the Anesthesiology Department.

The research team worked together to create two surveys, one for CRTs and one for investigators, to ensure the questions included were representative of CRT and investigator experiences. The questions were circulated to the whole team until a consensus was reached on the final questions to be included on the survey. The questions centered on the experiences of both groups in the program, attitudes about the program, and how it helped each group achieve their research and professional goals. There were five questions on the investigator survey and seven on the CRT survey (see the Appendix). The available responses, based on a five-point Likert Scale, were “strongly agree,” “agree,” “neither agree nor disagree,” “disagree,” and “strongly disagree.” There was a blank comment section at the end of each survey for participants to offer feedback, criticism, and reflect on their attitudes towards the program. Subjects were told that the survey was for feedback on the program not for research purposes. The surveys were also anonymous, to avoid bias in responses.

Inclusion criteria for CRTs were those who had been part of the program from January 2016 until May 2022. For investigators, it was those who currently or previously had utilized one or more CRTs to help conduct their research in some capacity (data collection, analysis, manuscript writing, or publication). The research leadership compiled and voted on a list of investigators deemed to have met these criteria. Exclusion criteria were any investigators who did not directly work with CRTs, or the investigators and CRTs assisting with this study. The CRT survey was distributed via a text link and the investigator survey was distributed via an email. Because the survey was anonymous, all eligible CRTs and investigators were sent one reminder to complete the survey via text or email, respectively.

To analyze survey responses, the comments were thematically coded [4]. Potential themes were discussed before distributing the survey and after the results were obtained. Themes were identified by the research team based on review of the responses using an iterative, consensus building process until agreement was reached. Additionally, the comments were coded as positive or negative, based on sentiment analysis, by one researcher and voted on for agreement by a second investigator [5]. The CRT and investigator surveys were analyzed separately, and five themes were identified for each survey. Data were also collected, based on historical departmental values, on the number of research grant approvals, research funding awarded, and CRT salaries each year since the start of the program. Data on research nurse salaries were collected from the NIH Department of Health and Human Services Clinical Center Nursing Department [6]. Data on clinical research coordinator hourly pay and job capabilities were collected from the Wake Forest University School of Medicine Clinical and Translational Science Institute Pricing Grid and website [7-8].

## Results

We received 20 responses (out of the n=29) from investigators yielding a 68.9% response rate. Out of the 20 responses, there were eight narrative comments yielding a 40% comment rate. We received 21 (out of n=22) responses from CRTs yielding a 95.4% response rate. Out of the 21 responses, six narrative comments were left yielding a 33.3% comment rate.

Investigator themes

Research Accuracy and Precision

**Figure 1:**
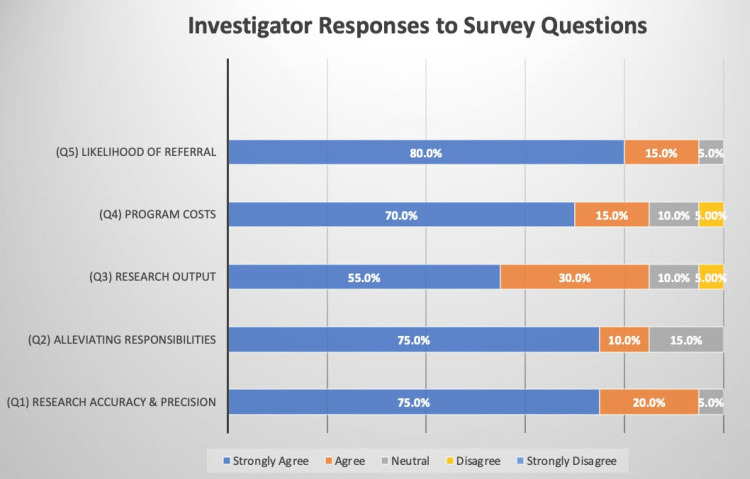
Investigator responses to the survey. Majority of respondents “Strongly agreed” to each of the seven questions.

Investigators’ perceived quality of research conducted by CRTs was assessed by the statement, “My research has been conducted accurately and precisely with the help of CRTs.” Ninety-five percent of investigators strongly agreed or agreed with the statement (Figure 1). 

Two comment excerpts were positively coded for this theme (Table 1).

**Table 1:** Summary of major themes and quotes identified in investigator and CRT survey responses relating to CRT program. CRT, clinical research technician

Investigator themes	Representative quotes
Research accuracy and precision	“Great team - very very good at their job” “They do a fantastic job and for the most part, require little guidance after adequately trained.”
Research output	"They seem happy to be involved, and I genuinely hope they get as much out of it as they seem to, because they're an invaluable asset to the department without whom I would probably not be conducting a randomized trial.” “The two studies I have been the PI for would have been significantly more difficult without the assistance of the CRTs.” “I had not been able to get manuscripts organized, written and submitted until several CRTs jumped in and accomplished most of this work. I have had 4 publications with their assistance.”
Alleviating responsibilities	“Easy and obvious answer for the busy clinician” “I can't say enough good things about the research technicians and how much it has decompressed my workflow. I am more able to engage with residents and fellows and focus on clinical duties without the added stress of running my studies.
Cost	“Cannot comment on the cost effectiveness as we do not know salaries of these individuals or where the funding comes from” “You should, however, better share the financial benefits of the program as many perceive it to be "red" or revenue neutral,”
Likelihood of referral	“I was one of the first to benefit from CRTs in our department and I am grateful for their assistance. I fully support the continuation of this program in our department.” “The CRTs are a wonderful merger of undergrad with post grad. This is part of translational research and development of future researchers and healthcare professionals.”
CRT themes	Representative quotes
Future career assistance	“The program lived up to all its expectations and beyond. The experience I gained will give me such a big head start going into medical school.” “This job had great flexibility and the understanding that it was a gap-year position for most of us to hopefully matriculate into medical school. That openness and expectation allowed us to really grow and be open in our intentions with this job (like leaving after a year or trying to get into projects and get our names on papers).” “This was the most important experience for me to have as a gap year student…I still use things I learned at this job as a medical student today!”
Physician career insights	“This job provided me a wealth of knowledge about the medical field, specialties, lifestyle…that will last a lifetime”. “It gave me so much perspective on what practicing actual medicine was like and helped me understand what I was getting into.”
Mentorship	“This job provided me…mentors that will last a lifetime.”
Likelihood of referral	“I cannot speak highly enough of this position and hope that it continues to grow to allow more future medical students and providers a great opportunity for their gap year.”
Other	“I was very happy to see the diversity grow and how that continues given how Wake Forest as a private institution isn't all that diverse.” “Great experience!”

Research Output

Research output was defined as the amount of research (i.e., number of studies) being conducted and any abstracts, manuscripts, or publications resulting from those research projects. Output was assessed by the statement, “My overall research output has increased since utilizing the help of the CRTs.” Eighty-five percent of investigators strongly agreed or agreed with the statement (Figure 1). Three comment excerpts were positively coded for this theme (Table 1).

Alleviating Responsibilities

Utilization of CRTs to help investigators focus on their other responsibilities was assessed by the statement, “I have been able to focus my efforts on clinical, educational, and/or administrative roles since I have started utilizing the CRTs to carry out the day-to-day responsibilities in my research.” Eighty-five percent of investigators strongly agreed or agreed with the statement (Figure 1). Two comment excerpts were positively coded for this theme (Table 1).

Costs

Perceived costs of using CRTs within the department were assessed with the statement, “The use of CRTs has provided the department a low-cost, high-yield alternative that aids in the overall research goals and accomplishments of the department.” Eighty-five percent of investigators strongly agreed or agreed with the statement (Figure 1). Two comment excerpts were negatively coded for this theme (Table 1).

Likelihood of Referral

Investigators’ attitudes towards and likelihood of referring the CRT program to other investigators or departments was assessed with the statement, “I would recommend the use of CRTs to help conduct research to other physicians/institutions.” Ninety-five percent of investigators strongly agreed or agreed with the statement (Figure 1). Two comment excerpts were positively coded for this theme (Table 1).

CRT themes

Future Career Assistance

**Figure 2:**
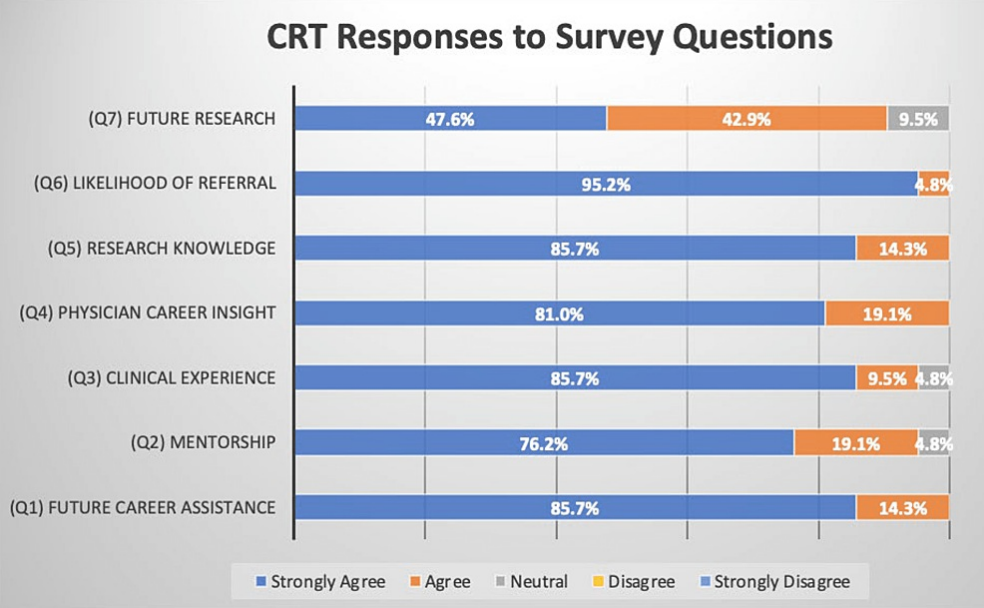
CRT responses to the survey. Majority of respondents “Strongly agreed” to each of the seven questions. CRT, clinical research technician

The CRTs’ perception of the program’s benefits and if the program helped them gain skills that could be utilized in a future healthcare profession was assessed with four statements. All of CRTs strongly agreed or agreed with the statement, “This program has/will help me matriculate into a healthcare graduate program” (Figure 2).

Ninety-five percent of CRTs strongly agreed or agreed with the statement “I gained hands-on clinical experience and direct patient contact” (Figure 2). One hundred percent of CRTs strongly agreed or agreed with the statement, “I have a greater understanding of research and the processes/requirements involved.” (Figure 2). Lastly, 90% of CRTs strongly agreed or agreed with the statement “I plan to participate in research throughout graduate school and as a provider” (Figure 2). Three comment excerpts were positively coded for this theme (Table 1).

Physician Career Insights

The knowledge gained by CRTs about a physician’s lifestyle was assessed with the statement, “I better understand the daily lifestyle of a provider and it gave me insight to what a future in the field may look like.” One hundred percent of CRTs strongly agreed or agreed with the statement (Figure 2). Two comment excerpts were positively coded for this theme (Table 1).

Mentorship

The access and ability to find mentors by working in the program was assessed with the statement, “This position gave me opportunities to interact candidly with physicians and find a mentor/mentors to help me throughout my gap year/application cycle.” Ninety-five percent of CRTs strongly agreed or agreed with the statement (Figure 2). One comment excerpt was positively coded for this theme (Table 1).

Likelihood of Referral

The CRTs’ attitudes towards the program and their likelihood of referring it to other gap-year students was assessed with the statement, “I would recommend this program for other students looking for a gap-year opportunity before starting a healthcare graduate program.” One hundred percent of CRTs strongly agreed or agreed with the statement (Figure 2). One comment excerpt was positively coded for this theme (Table 1).

Other

**Figure 3:**
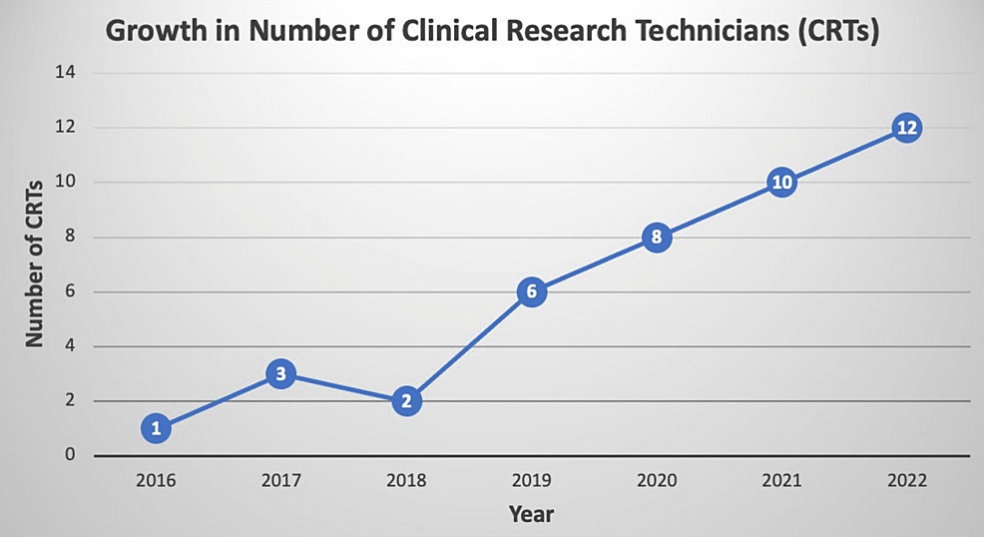
Growth in the number of CRTs per year from 2016 to 2022. CRTs, clinical research technicians

For comments that did not fall into one of our selected themes we compiled an “Other” category. Two comment excerpts were positively coded for this theme (Table 1). Each year the program added CRTs. Growth in the size of the CRT program is shown in Figure 3 and 100% of CRTs have been accepted into the health profession program they were seeking. 

**Figure 4:**
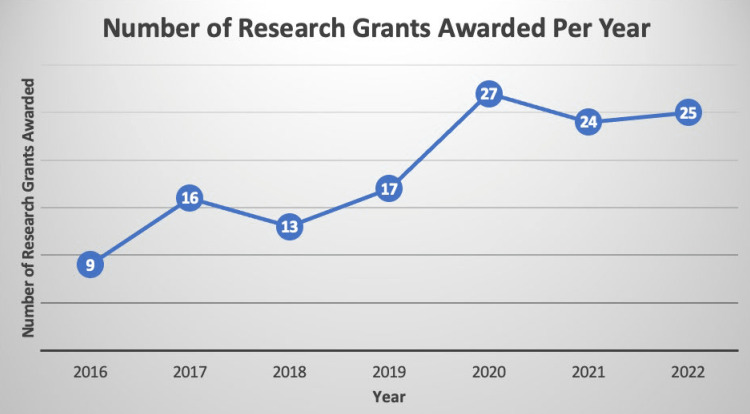
Growth in the number of grants awarded per year from 2016 to 2022.

**Figure 5:**
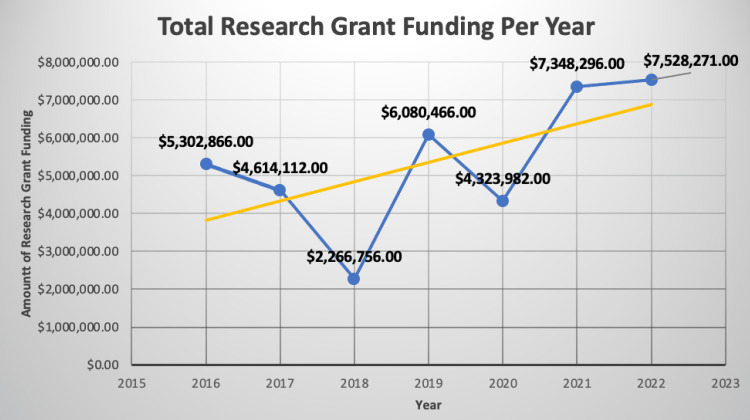
Growth in research grant funding per year from 2016 to 2022. Yellow line is a linear regression fit of the data.

Grant approvals and funding have also steadily increased over the lifespan of the program (Figures 4-5).

The hourly cost of using a clinical research coordinator at the institution is $60 compared to the hourly cost of using a CRT which is $20 [7]. The average clinical research nurse salary ranges from $84,000 to $118,000 and the current CRT salary is approximately $35,000 [6].

## Discussion

**Figure 6:**
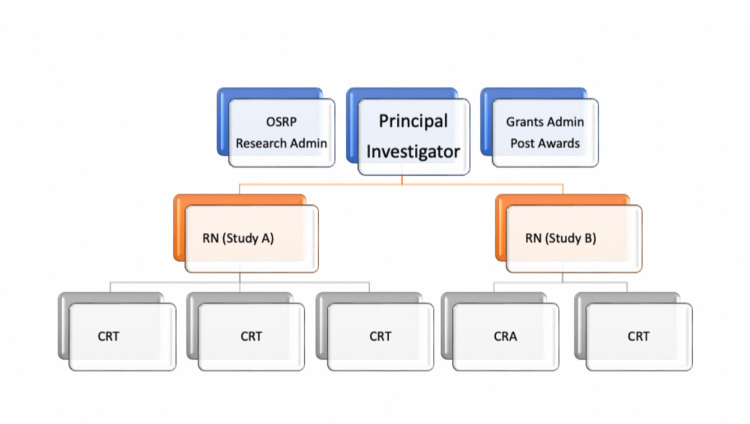
Research mechanistic at Wake Forest University School of Medicine Anesthesiology with the CRT-centered mini-clusters of teams consisting of CRTs, CRAs, and RNs. OSRP, Office of Sponsored Research Program; CRT, clinical research technician; CRAs, clinical research associates; RNs, research nurses

We believe that the CRT program has been mutually beneficial for both students and research investigators. Overall, investigators and CRTs have positive attitudes towards the program as demonstrated by the survey responses. As noted above, negative comments were rare and confined to ambiguity of the program costs and sources of funding. Increasing financial transparency about the program and the research money supported by the program could help alleviate these negative attitudes. Furthermore, it might be useful to publicize that the hourly rate of a CRT is a third of the hourly rate of a study coordinator [6]. While the current CRT salary is less than half the average clinical research nurse salary, the clinical research nurses do form the backbone of the structure and organization, and a nurse typically leads more than one clinical research trial in direct coordination with the clinical principal investigator (PI) and a cohort of CRTs (Figure 6). 

Our institution offers a Study Coordinator Pool, which provides investigators with research personnel to help conduct clinical research studies. The description of this program claims that the trained research staff allows investigators to conduct research quickly and lessen the administrative burden of having to train and manage a research team. We analyzed the job description of a “Study Coordinator” to see how it compared to that of a CRT. We found that the only major differences in job descriptions were that Study Coordinators were trained in IRB and regulatory management (i.e., assembling and maintaining regulatory binders, submitting protocols and amendments to the IRB) and had additional clinical coordination capabilities (i.e., communicating with external sponsors to schedule monitoring visits, specimen draw/processing, and receiving inventory study supplies). However, CRTs shared many of the clinical job responsibilities such as prescreening, recruitment, consenting, data collection, adverse event and serious adverse event reporting, and subject-related communications.

The support structure for this program includes leadership from a Vice-Chair of Research, a senior research nurse manager, four other research nurses, one to two research fellows, and an additional two clinical research associates (clinical research associates, CRAs; medical students or medical school graduates spending research years in the department). The organizational strategy is based on cohorts of three to four CRTs paired with one registered nurse (RN) with or without a CRA for each clinical research trial. Although we primarily report on the roles and experiences of CRTs and investigators, we wanted to acknowledge the support structure for this program provided by other personnel.

In our model, we utilize the head research nurse, who is responsible for the instituitional review board (IRB) and regulatory management while the remaining nurses and CRTs manage majority of the clinical coordination responsibilities. Often the protocols in the department require more personnel to do data collection, subject screening, consenting, and clinical responsibilities than regulatory management. Essentially our department has created a CRT pool, where CRTs are available to all anesthesiology investigators in need of assistance for research. These services come at no cost to them, but rather, the department covers the cost. This model also benefits CRTs because they are able to work with multiple investigators (providing opportunities for multiple mentors/educational experiences) and they are working on multiple research trials (expanding their understanding of various projects, areas of research, and types of research).

Mentorship is important in the context of medical school, as it can impact personal development, career guidance, career choice, research productivity, publication, and grant success. Yet many medical students report not having a mentor [9]. Our program provided a space for students and investigators to make these connections, with over two-thirds reporting they had opportunities to work with and find a mentor to help with their application process. The CRTs, likewise, reported feeling prepared to take on the challenges of medical school with over three-fourths reporting that the program helped them gain clinical experience and feel prepared to enter medical school.

At an academic institution, investigators seek out opportunities to conduct research for a variety of reasons, including promotion and/or a desire to contribute knowledge and advancements to the field of medicine. Barriers to conducting research can include other clinical responsibilities as well as lack of time to conduct research [10]. Our program demonstrates success in helping alleviate these barriers as over two-thirds of investigators agreed that using CRTs allowed them to focus on other clinical, educational, and administrative commitments. Additionally, using clinical research coordinators can help increase research output and publications by over 50% [11]. Our investigators agreed with this sentiment, with over three-fourths agreeing that their research output has increased since utilizing CRTs. However, this could also be due to the support of other research team members such as research nurses, medical students, research associates, postdoctoral fellows, and other physicians.

We also aimed to determine if the program aids gap-year students in matriculating to medical school and being prepared for clinical interactions. Research has shown that participating in a research year can add to pre-medical students’ medical school applications [12]. Additionally, students taking one or more gap years prior to medical school tend to have lower levels of burnout than their counterparts who do not [13]. We defined the success of this program by the percentage of CRTs who matriculated into their preferred graduate health programs (medical school or physician assistant school), as indicated during their interview for the CRT program. To date, this has been 100%. There was one CRT who was waitlisted and completed a second year in the program while reapplying and ultimately matriculating to medical school at the end of two years in the program. Combined with the positive survey responses relating to skills and knowledge carried over from this job to medical school, this leads us to believe this program is valuable to gap-year students. It is possible that the positive response from CRTs is due to the fact that every CRT matriculated into a graduate program of their choice by the end of their time in the program. It is also plausible that the CRTs hired for the job were already talented graduate health program candidates and would have been admitted regardless of their time spent in the CRT program. However, CRTs expressed they felt like this program benefited them, regardless of the causality. The CRTs’ feelings about the program and the candid feedback can be used to continue improving and growing the program. Positive experiences mean more referrals of the program in future years, which makes this a self-sustaining system as more and more students elect to take gap-years.

Our secondary objectives were to review the growth in research funding and grant approvals since utilizing CRTs. While the growth in the number of CRTs cannot be causally correlated with the growth in research grants, we found that both have linearly increased from 2016 to 2022 (Figures 3-4). We acknowledge that indeed the causality could be reversed, in that CRT growth could have reflected grant success, which offered a funding source. However, investigator-initiated and departmentally funded projects also increased, so we do not believe that growth in funding is the sole explanation of growth in the CRT program.

Limitations to our research include that we did not analyze the roles and impact of additional members of the research team such as research nurses, clinical research associates, medical students, and research fellows. In addition, we did not attempt to identify any other similar programs at other institutions. The survey questions were not created by a psychometric expert and may be perceived as leading. This could result in biased responses and limit the validity of conclusions. Additionally, the small number of comments received on the survey makes it difficult to draw definitive conclusions. Because the research team included the department administrators for the CRT program, this could have also biased the interpretation of the results. Additionally, research participants were not informed that the survey was for research purposes which may have affected the responses, although it is impossible to ascertain if blinding would lead to more positive or negative reviews. Further research could investigate the longer-term career trajectories of CRT graduates, for example whether they sought academic careers in the future. Additionally, a study examining departments at the same institution who only use research nurses versus CRTs could prove beneficial.

## Conclusions

There are few structured, clinical research, gap-year programs for premedical students, and there is no published research on how these programs are built and how successful they are. Programs like ours could serve as a new educational tool for premedical students as well as an important research infrastructure resource for hospitals and medical schools. This type of program could be especially beneficial at institutions where the pre-health, gap-year student community remains untapped as a resource. By helping premedical students and healthcare institutions understand the value of programs like ours, we might encourage them to utilize a similar model.

## Appendices

Clinical Research Technician (CRT) Program Opinion Survey - CRTs:

1. This program has/will help me matriculate into a healthcare graduate program.

2. This position gave me opportunities to interact candidly with physicians and find a mentor / mentors to help me throughout my gap-year / application cycle.

3. I gained hands-on clinical experience and direct patient contact.

4. I better understand the daily lifestyle of a provider and it gave me insight to what a future in the field may look like.

5. I have a greater understanding of research and the processes / requirements involved.

6. I would recommend this program for other students looking for a gap-year opportunity before starting a healthcare graduate program.

7. I plan to participate in research throughout graduate school and as a provider.

Clinical Research Technician (CRT) Program Opinion Survey - Investigators:

1. My research has been conducted accurately and precisely with the help of the CRTs.

2. My overall research output has increased since utilizing the help of the CRTs.

3. I have been able to focus my efforts on clinical, educational, and/or administrative roles since I have started utilizing the CRTs to carry out the day-to-day responsibilities in my research.

4. The use of CRTs has provided the department a low-cost, high-yield alternative that aids in the overall research goals and accomplishments of the department.

5. I would recommend the use of CRTs to help conduct research to other physicians / institutions.
